# Effect of aerobic exercise training on cardiometabolic risk factors among professional athletes in the heaviest-weight class

**DOI:** 10.1186/s13098-015-0071-y

**Published:** 2015-09-17

**Authors:** Jianjun Guo, Yanmei Lou, Xi Zhang, Yiqing Song

**Affiliations:** Centre for the Youth Sport Research and Development, China Institute of Sport Science, Tiyuguan Road, Beijing, China; Department of Health Management, Beijing Xiao Tang Shan Hospital, Beijing, China; Department of Epidemiology, Richard M. Fairbanks School of Public Health, Indiana University, Indianapolis, IN USA

**Keywords:** Aerobic exercise, Cardiometabolic risk factors, Athletes, Metabolic syndrome

## Abstract

**Background:**

High prevalence of metabolic diseases among young professional athletes with large body sizes has raised growing attention. However, few studies specifically examined whether additional aerobic exercise provides cardiometabolic beneficial effect among these young athletes under regularly intensive strength training.

**Objective:**

We conducted a pilot trial to evaluate the effects of aerobic exercise on overall metabolic syndrome (MetS), individual MetS components, and aerobic capacity among metabolically unhealthy athletes in the heaviest-weight class.

**Methods:**

Forty-nine professional athletes aged 15–30 years had large body weights (mean weight of 131 ± 15.5 kg and 108 ± 15.8 kg and mean BMI of 39.4 ± 4.7 kg/m^2^ and 36.4 ± 5.1 kg/m^2^ for 26 men and 23 women, respectively). They completed a supervised moderate intensity (maximal heart rate: 140–170 beats/min for 30–70 min/day) aerobic exercise training for 12 weeks. We collected and measured metabolic parameters and aerobic capacity for all participants before and after 12 weeks of aerobic exercise training.

**Results:**

At baseline, 42 (86 %) of all 49 metabolically abnormal athletes were diagnosed as MetS according to the NCEP ATP III criteria (≥3 MetS components). After aerobic exercise training, 30 % (13/42) of MetS individuals tended to become free of MetS (<3 MetS components), decreasing the prevalence of MetS by 30.4 % (from 17 to 10) in women and 23.1 % (from 25 to 19) in men (P = 0.001). All individual components of MetS, including fasting glucose levels, lipid profile, and blood pressure, were also significantly improved (all P-values <0.05). Overall and central obesity indexes, including BMI, waist circumference, Waist-hip ratio, and abdominal fat ratio, were significantly decreased in men whereas only overall adiposity indexes, such as BMI and body fat percentage, were significantly reduced in women. Also, participants’ aerobic capacities were also significantly enhanced with longer running distances and decreased heart rates (all P-values <0.05).

**Conclusions:**

Our pilot trial showed that moderate intensity aerobic exercise effectively improved cardiometabolic parameters in metabolically unhealthy professional athletes with routinely intensive strength training. Its long-term cardiovascular effects will be evaluated by future randomized controlled trials with well-designed exercise modalities.

## Background

Cardiometabolic risk factors for diabetes mellitus and cardiovascular diseases (CVDs) have been associated with cardiovascular morbidity and mortality in the general population [1]. More recently, several reports found that young, active, and seemingly “healthy” professional athletes were not free of cardiometabolic risk, especially those with large body sizes [2, 3]. American football players with large body sizes were found to have 52 % higher risk of heart disease mortality than individuals from general population [4]. Consistently, our previous survey found that 261 Chinese professional athletes of strength sports in the heaviest-body- weight-class had much higher prevalence of metabolic risk factors and metabolic syndrome (MetS) as an entity than their counterparts at all other weight groups [5]. Thus, it becomes imperative to develop and implement effective preventive strategies targeting cardiovascular health among young athletes in the heavy-weight class.

Exercise has been widely accepted as an efficient preventive strategy for improving cardiometabolic health [6]. Although cardiometabolic benefits of exercise have been well documented, the relative effects of different types of exercise (aerobic or anaerobic exercise) remain uncertain due to sparse data [7]. A meta-analysis for 12 randomized clinical trials of aerobic exercise in patients with type 2 diabetes found that aerobic exercise had greater effects on reduction of the glycosylated hemoglobin, BMI, maximum heart rate and rise of VO_2peak_ than resistance exercise [8]. We thus hypothesized that aerobic exercise may provide additional beneficial cardiovascular effects on its individual components of MetS and aerobic capacity among professional athletes who routinely undergo intensive strength training, most of being anaerobic exercise.

We conducted a pilot trial of 12-week moderate intensity aerobic exercise intervention among young active professional athletes in the heaviest-weight class, who were metabolically unhealthy (at least one of four MetS components with exception of abdominal obesity).

## Methods

### Research design and participants

This 12-week, one-arm trial targeted metabolically abnormal athletes in heaviest-weight class in China (from Hebei, Liaoning, Jilin, and Shanxi provinces). In total, 82 athletes in the heaviest-weight class were at the Sports Institute for training and routinely examined for cardiometabolic risk factors. They were screened by the initial survey for eligibility. Finally, a total of 49 athletes aged from 15 to 30 years, 26 men and 23 women, consent to participate in this trial from July 2006 to December 2008. Of them, 36 were weightlifting athletes, 10 were judo athletes, and 3 were track and field throwing athletes. All 49 participants included in this study were currently professional athletes and in their off-season periods for physical preparation. Among them, 13 were elite athletes (international level) and 36 athletes were national level. Generally, they received 4–5 h professional training per day, 6 days per week. On average, each participant had received 7-year training. The participants were instructed to remain their regular training and lifestyle during the study. This trial protocol was reviewed and approved by the Institutional Review Board of China Institute of Sport Sciences, Beijing, China and written informed consents were obtained from all athletes who participated in the trial.

### Anthropometric measurements

Anthropometric examination, physical examination, measure of blood lipid profile, and aerobic capacity test were conducted for each participant according to the study protocol and the corresponding information were collected before and after the 12-weeks exercise training. Height, weight, waist circumference, hip circumference, sitting blood pressure, and body composition were measured by professional physicians as previously described in details [5]. Briefly, height, waist circumference, hip circumference in centimeter, and weight in kilogram were measured with light clothes. Waist circumference was measured at the midpoint between the inferior costal margin and the superior border of the iliac crest on the midaxillary line and hip circumference was measured at the maximum extension of the buttocks. Waist-hip ratio (WHR) is anthropometric indices of central adiposity which was calculated by dividing the waist circumference by the hip circumference. BMI was estimated through the equation of weight/height^2^ (kg/m^2^). Abdominal fat ratio was calculated by dividing the abdominal fat volume by total abdominal volume. Blood pressure (BP) was measured at least twice using a calibrated conventional mercury sphygmomanometer. For each participant, an average value of 2 blood pressure measurements at a 3-min interval was used as the final BP measurement. After 10 min of rest, the right-arm SBP and DBP were recorded in the supine position for every participant seated in a quiet room. Body fat percentage was measured by bioelectrical impedance analysis (DSM-BIA) using the In-Body (3.0) body composition analyzer, which is widely used in large-scale population studies because of its reasonable quality, simple technology, and affordable price [9].

### Laboratory and biochemical measurements

After 10–12 h fasting, and avoiding of smoking, drinking alcoholic beverages and coffee prior to the scheduled appointment, participants sat at ease and had a rest for 5 min to prepare for the measurements. All athletes had their training for 6 days per week, from Monday to Saturday, and were at rest on Sunday. All fasting blood samples were collected on Monday mornings so that athletes had no exercise training for at least 36 h prior to their blood collections. Venous blood samples were drawn from an antecubital vein into potassium oxalate/sodium fluoride anticoagulant tubes by using standard venipuncture methods.

Plasma glucose was determined by hexokinase method, with intra- and inter-assay coefficients of variations (CVs) of less than 2.5 and 3.5 %, respectively. Enzymatic colorimetric assay (Hitachi 7170A, Japan) was used for the measurements of triglyceride (TG), high density lipoprotein cholesterol (HDL-C), and low-density lipoprotein cholesterol (LDL-C). The intra- and inter-assay CVs were less than 1.5 and 3.0 %, respectively. Blood lactate was assayed according to an enzymatic method as described previously [10]. Blood samples for lactate measure were collected from each athlete at 5 min after his/her 12-min running test.

### Definition of MetS

The MetS is diagnosed according to the National Cholesterol Education Program Adult Treatment Panel III (NCEP ATP III) criteria in 2003 [11], which was defined by the presence of three or more of the five individual components listed below: (1) waist circumference for abdominal obesity: men ≥102 cm or women ≥88 cm; (2) elevated triglycerides (≥1.7 mmol/L); (3) lower HDL-C (men <1.04 mmol/L and women <1.03 mmol/L); (4) elevated BP (systolic ≥130 mmHg and/or diastolic ≥85 mmHg); and (5) elevated fasting glucose [≥6.1 mmol/L and (or) diabetes]. Participants in this pilot trial were all metabolically abnormal with at least one of the four MetS components (with exception of abdominal obesity).

### Assessment of aerobic capacity

We administered Cooper 12-min running test to indirectly evaluate the capacity of aerobic exercise, which is widely adopted by researchers because of its effectiveness and simple availability [12, 13]. According to the standard guideline of 12-min running test for China’s college students [14], the pass levels are 1600–1900 m for women and 2000–2300 m for men. Good and excellent levels are 2000–2300 m and ≥2400 m for women and 2400–2700 m and ≥2800 m for men, respectively. During 12 min, the athletes tried their best to run around the 400-m athletic field under the supervision of a professional coach. The total distance of 12 min’ running was recorded for each athlete. The test for everyone was conducted twice in every other day, if the difference was greater than 5 %, the test would be redone on the next day, and then the mean of the two measurements was calculated for analyses. Heart rates during exercise and 5 min after exercise were assessed by POLAR^®^ sports heart meter.

### A supervised moderate intensity aerobic exercise training

In their routine strength training, the intensity, frequency, and time of training varied across the types of strength sports. Routinely, they have taken a 2–3 h vigorous strength training (rating perceived exertion RPE: 17–20) twice daily for 6 days per week (from Monday to Saturday). After the regular morning and afternoon strength training, an additional 30 to 70-min aerobic training per day was conducted by every athlete in this trial. A 12-week training of moderate aerobic exercise was conducted among all participants.

The appropriate fat-reducing exercise intensity proposed by American College of Sports Medicine (ACSM) is 50–70 % VO_2max_ [15]. The participants’ heart rates were controlled between 140 and 170 beats/min, equivalent to a moderate intensity fat reduction exercises at the intensity between 50 and 60 % VO_2max_ according to the conversion function between the heart rate and maximal oxygen uptake proposed by Astrand in 1960 [16]. The time of aerobic exercise was determined according to the actual intensity of aerobic exercise. Thirty minutes aerobic training was required to keep participants’ heart rates ≥160 beats/min; if their heart rates were between 140 and 150 beats/min, 70 min exercise was required to ensure an isometric exercise. The exercise types included running, jogging, bicycle pedaling, and swimming. The whole process of the aerobic exercise training was supervised by qualified physical professionals to ensure the required intensity and duration.

### Statistical analysis

The sample size was calculated based on measurements of HDL-C given a power of 90 % and a type I error probability of 0.05. A sample size of 10 athletes could achieve a power to detect a clinically significant effect on HDL-C by 0.15 ± 0.13 mmol/L [17]. Given a relatively high rate of physical injuries observed among our professional athletes who received both strength training and aerobic exercise training, we allow for a 20 % maximal withdrawal rate and noncompliance. As a result, at least 40 athletes were needed to ensure sufficient statistical power. Finally, we included a total of 49 athletes in this trial.

All continuous variables were presented as the means ± standard deviations (SDs) for normal distribution and median and quartiles for abnormal distribution, respectively. Categorical variables were described as count (frequency). The changes of demographic and biochemical factors between pre- and post-measurements were compared using matched t-tests or related-samples Wilcoxon signed rank tests appropriately. The prevalence of individual abnormal MetS components and the MetS (≥3), metabolically abnormal (≥2), or metabolically healthy (none with the exception of obesity) were determined at baseline and after the trial. Chi square tests were used to compare the transitions in the point prevalence of MetS and individual MetS component between baseline and end of the trial for men, women, or both, respectively. The modifying effect by extreme adiposity (BMI <40 kg/m^2^ and ≥40 kg/m^2^) was tested by performing subgroup analysis. Level of significance was two-tailed P < 0.05 for all statistical analyses. All data analyses were performed using SPSS version 20 (SPSS Inc., Chicago, IL, USA).

## Results

### Basic characteristics of the study participants

As summarized in Table 1, all 49 young athletes (26 men and 23 women) in the heaviest-weight class with a median age of 20 years (range 15–30) completed this 12-week aerobic exercise training (30–70 min/day) without dropout or withdrawal. These athletes had a mean weight of 131 kg for men and 108 kg for women (BMI range 27.2–49.5 kg/m^2^) and a mean BMI of 39.4 kg/m^2^ for men and 36.4 kg/m^2^ for women. As expected, men and women apparently differed in the demographic and anthropometric measurements, such as age, BMI, and waist circumference. All participants were metabolically abnormal because they had at least one MetS component with exception of abdominal obesity, including, hypertension, hypertriglyceridemia, low HDL and fasting hyperglycemia.

**Table 1:** Comparison of weight, body composition and blood lipid of 49 athletes of different genders at pre-intervention and post-intervention

Gender	Measurements	Pre-intervention	Post-intervention	P-values
Women, N = 23	BMI (kg/m^2^)	36.4 ± 5.1	35.3 ± 4.5	<0.0001
	Body fat percentage (%)	33 ± 0.05	32 ± 0.05	0.02
	Abdominal fat ratio	0.94 ± 0.09	0.95 ± 0.08	0.55
	Waist circumference (cm)	104.0 ± 10.7	103.8 ± 10.6	0.13
	WHR	0.92 ± 0.11	0.92 ± 0.11	0.5
	SBP (mmHg)	120 (115, 130)	120 (110, 124)	0.04
	DBP (mmHg)	80 (70, 90)	80 (70, 86)	0.03
	TG (mmol/L)	2.64 ± 0.57	1.97 ± 0.66	<0.0001
	TC (mmol/L)	4.79 (4.08, 5.68)	4.79 (4.06, 5.26)	0.01
	HDL-C (mmol/L)	1.13 (0.86, 1.35)	1.35 (1.07, 1.63)	<0.0001
	LDL-C (mmol/L)	2.56 ± 0.74	2.43 ± 0.76	0.06
	FPG (mmol/L)	5.46 (4.87, 6.21)	5.16 (4.57, 5.73)	<0.0001
	12-min running test (m)	1711 ± 132	1995 ± 226	<0.0001
	Heart rate (beats/min)^a^	122.4 ± 11.2	114.4 ± 14.5	<0.0001
	Blood lactate (mmol/L)^a^	1.57 ± 0.71	1.38 ± 0.62	0.17
	Components of MetS, n (%)			0.03
	With 1 component	0 (0.0)	5 (2.2)	
	With 2 components	6 (26.1)	8 (3.5)	
	With ≥3 components	17 (73.9)	10 (43.5)	
Men, N = 26	BMI (kg/m^2^)	39.4 ± 4.7	38.1 ± 4.5	<0.0001
	Body fat percentage (%)	30 ± 0.05	29 ± 0.05	0.17
	Abdominal fat ratio	1.03 ± 0.09	0.99 ± 0.06	0.01
	Waist circumference (cm)	114.8 ± 10.5	114.3 ± 10.5	<0.0001
	WHR	1.02 ± 0.15	1.01 ± 0.15	0.08
	SBP (mmHg)	140 (130, 150)	135 (125, 147)	0.03
	DBP (mmHg)	95 (85, 100)	90 (80, 95)	0.003
	TG (mmol/L)	5.07 ± 1.99	4.17 ± 1.46	0.03
	TC (mmol/L)	5.37 (4.87, 5.91)	5.19 (4.58, 5.77)	0.001
	HDL-C (mmol/L)	1.09 (0.86, 1.36)	1.24 (1.02, 1.38)	0.001
	LDL-C (mmol/L)	3.11 ± 0.49	2.82 ± 0.50	<0.0001
	FPG (mmol/L)	5.81 (5.18, 6.89)	5.21 ± 0.61	<0.0001
	12-min running test (m)	1892 ± 260	2259 ± 256	<0.0001
	Heart rate (beats/min)^a^	123.7 ± 9.7	118.1 ± 12.3	<0.0001
	Blood lactate (mmol/L)^a^	1.46 ± 0.64	1.22 ± 0.53	0.06
	Components of MetS, n (%)			0.64
	With 1 component	0 (0.0)	2 (7.7)	
	With 2 components	1 (3.8)	5 (19.2)	
	With ≥3 components	25 (96.2)	19 (73.1)	

All continuous variables were presented as the mean ± SD and compared by paired sample t-tests for normal distributed variables, and median (lower quartile, upper quartile) and related-samples Wilcoxon signed rand tests for variables with abnormal distribution. Categorical variable was presented as n (%) and compared by fisher’s exact test

^a^The measurements were conducted 5 min after the training of aerobic exercise

### Effects of aerobic exercises on body fatness

After this short-term moderate intensity aerobic exercise intervention, body fatness of athletes was moderately and significantly decreased. On average, body weights were reduced by 3.7 kg (P < 0.0001) with a reduction of 6 % (95 % CI 0.2–1 %) in body fat percentage (P = 0.006) in these young active athletes. There was a significant sex difference in the effects of aerobic exercise on body weight indexes. Overall and central obesity indexes, including BMI, waist, WHR, and abdominal fat ratio, were significantly decreased in men whereas global obesity indexes, including BMI and body fat percentage, were only significantly reduced in women.

### Effects of aerobic exercises on levels of metabolic biomarkers and aerobic capacity

All other individual MetS-related parameters, including levels of BP, triglyceride, HDL, and fasting glucose, were also significantly improved after a 12-week aerobic exercise (all P-values <0.05). Levels of TC, TG, and LDL-C were significantly decreased in both men and women athletes (all P-values <0.05) while HDL-C levels were significantly elevated by approximately 0.22 mmol/L in women (P < 0.0001) and 0.15 mmol/L in men (P = 0.001).

Aerobic exercise had a significant effect on aerobic capacity as a surrogate of combined physical and cardiorespiratory fitness for both male and female athletes. For women, there was significant 8 beats/min of heart rate measured at 5 min after exercise slower than at baseline (from 122 ± 11 beats/min to 114 ± 15 beats/min) and a 284 m in the exhausted running distance in 12 min (from 1711 ± 132 m to 1995 ± 226 m) higher at baseline than after the trial. The improvement of aerobic capacity were also found in men, from 1892 m (SD 260 m) at baseline to 2259 m (SD 256 m) and from 124 beats/min (SD 9 beats/min) to 118 beats/min (SD 12 beats/min) for heart rate after running (all P-values <0.0001). Lower levels of blood lactate after aerobic exercise indicated an enhanced physical recovery, although the change was marginally significant in men (P = 0.06).

### Effects of aerobic exercises on MetS transition

**Fig. 1:**
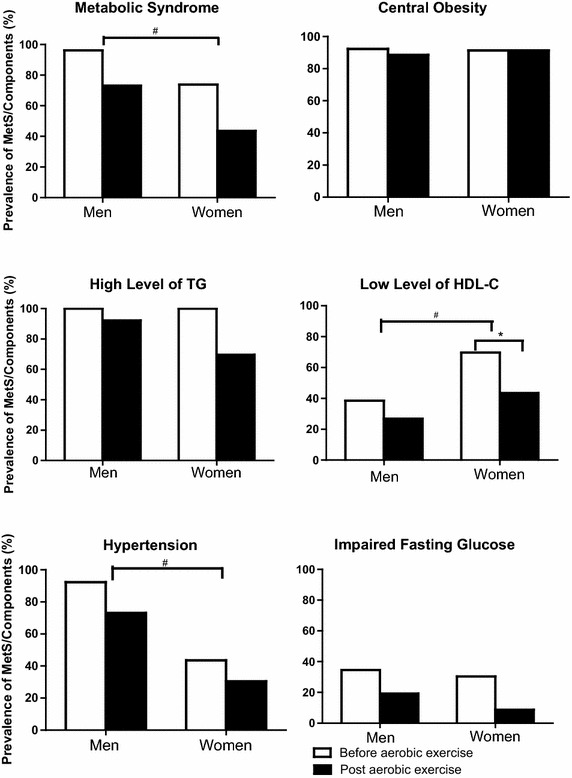
The sex-stratified prevalence of metabolic syndrome and its components before and after aerobic exercise treatment. The *empty bars* represented the pre-treatment values and the *black bars* represented the post-treatment values. Metabolic syndrome and its individual factors were defined by NCEP ATP III. The cutoff points are as follows: waist circumference for central obesity: men ≥102 cm, women ≥88 cm; hypertension: SBP/DBP ≥130/85 mmHg; FPG ≥6.1 mmol/L, and (or) diabetes; TG ≥1.7 or HDL-C <1.04 mmol/L (men) or 1.30 mmol/L (women). *P value compared the difference of the prevalence of participants having low levels of HDL-C at pre- and post-training in women; ^#^P-values compared sex differences of the prevalence of metabolic syndrome and its components were <0.05

In all, the overall prevalence of MetS decreased from 96.2 to 73.1 % in men and from 73.9 to 43.5 % in women after aerobic exercise training (P = 0.004) shown in Fig. 1. After aerobic exercise training for 12-week, 7/17 women and 6/25 men athletes with MetS (≥3 MetS components) at baseline transitioned to metabolically healthy (<3 MetS components), decreasing the prevalence of MetS by 30.4 % (from 17 to 10) in women and 23.1 % (from 25 to 19) in men (P = 0.001) as shown in Table 1. The prevalence of each of five individual MetS components, including central obesity, hypertriglyceridemia, low HDL levels, hypertension, and impaired fasting glucose, tended to be reduced by aerobic exercises in both men and women. However, there was only statistical significance for the decreased prevalence of low HDL-C prevalence from 69.6 % at baseline to 43.5 % at the end of training among women (P = 0.03).

### Effect modifications by BMI

These young active athletes may respond differentially to aerobic exercise in terms of metabolic parameters based on their levels of extreme obesity. Our subgroup analysis stratified by two groups (BMI <40 kg/m^2^ and BMI ≥40 kg/m^2^) as shown in the Table 2. Overall, the effects of aerobic exercise training on BP were more pronounced in the high BMI group than the low BMI group. Specifically, the decrement of SBP in high BMI group was fourfold higher (P = 0.007), and the DBP change was twofold higher in high BMI group than those of low BMI group (P = 0.09). Basal SBP and DBP between athletes with BMI <40 kg/m^2^ vs. those with BMI ≥40 kg/m^2^ were 128.9 vs. 140.8 mmHg (P = 0.02) and 85.2 vs. 83.1 mmHg (P = 0.06), respectively. Not all the SBP/DBP values in the low BMI group were normal. We further performed an ANCOVA test adjusted for baseline BP and found that the SBP lowering effect by exercise was modified by baseline SBP values. After aerobic exercise training, the response of post-exercise heart rate at 5 min was slowed by 7 beats/min (P = 0.008) in the high BMI group compare with the low BMI group. None of the differences of other measures between athletes with BMI ≥40 kg/m^2^ and those with BMI <40 kg/m^2^ reached statistically significant.

**Table 2:** Measurement changes (post-treatment minus pre-treatment) by different baseline

Measurements^a^	Baseline BMI		
	<40 kg/m^2^, N = 33	≥40 kg/m^2^, N = 16	P-values
Body fat percentage (%)	0.0009 ± 0.04	−0.05 ± 0.08	0.03
Abdominal fat ratio	0.0009 ± 0.04	−0.05 ± 0.08	0.03
Waist circumstance (cm)	−0.35 ± 0.73	−0.51 ± 0.77	0.45
WHR	0.0009 ± 0.01	0.01 ± 0.01	0.65
SBP (mmHg)	−1.82 ± 4.85	−7.56 ± 7.01	0.01
DBP (mmHg)	−1.97 ± 4.33	−4.31 ± 4.85	0.09
TG (mmol/l)	−0.89 ± 1.71	−0.58 ± 1.19	0.51
TC (mmol/l)	−0.11 ± 0.27	−0.21 ± 0.17	0.18
HDL-C (mmol/l)	0.21 ± 0.38	0.21 ± 0.45	0.95
LDL-C (mmol/l)	−0.18 ± 0.3	−0.29 ± 0.35	0.29
FPG (mmol/l)	−0.44 ± 0.61	−0.76 ± 0.79	0.12
12-min running test (m)	309 ± 190	367 ± 218	0.35
Heart rate (beats/min)	−4.3 ± 9.2	−11.8 ± 7.8	0.01
Blood lactate (mmol/l)	−0.22 ± 0.65	−0.22 ± 0.59	0.98

BMI classes among All 49 athletes

^a^Continuous variables were presented as the means ± standard deviations; the P values were calculated by using t-test or Mann–Whitney U test

## Discussions

Our pilot trial of 49 metabolically unhealthy athletes in the highest weight class has shown that 30–70 min/day moderate intensity aerobic exercise for 12 weeks elicited a significantly meaningful improvement in levels of conventional cardiometabolic risk factors and exercise capacity. Significantly, aerobic exercise training for 12 weeks resulted in a 20 % increment of blood HDL-C levels and nearly 24 % reversion from MetS to MetS-free status. Aerobic exercise training also improved exercise capacity as well as physical or cardiorespiratory fitness among these young athletes in heaviest-weight class. Despite its short-term and relatively small sample sizes, this pilot trial provided some encouraging empirical data that support the tremendous potential to implement aerobic exercise programming into professional athletes to prevent MetS-associated cardiovascular outcomes, primarily due to their large body size and fatness.

MetS is defined by a constellation of risk factors of cardiovascular disease, which might contribute to about twofold increase in the risk of developing CVDs and fivefold increase in the risk of type 2 diabetes mellitus in future among general population [18]. Previously, elite athletes were always identified as the symbol of physical health because of their young ages, heavy training loads, large cardiorespiratory capacity, and regular participation of competitions during their sports careers [19, 20]. However, in 1994, a study of retired National Football League players found a 52 % greater risk of cardiovascular death in linemen with large body weights than the general population and 3 times higher risk of dying from cardiovascular diseases than non-linemen [21]. Recently, high incidences of obesity, metabolic syndrome, and other cardiovascular risk factors were also found in young collegiate football linemen [22–24]. Similarly, our previous study reported a MetS prevalence of 89 and 47 % among male and female Chinese active strength athletes in the highest weight class compared with a prevalence of 18 and 0 % in their respective counterparts at other weight classes [5].

MetS is increasingly present in young professional athletes with large body weights and it could greatly increase risk of MetS-associated chronic diseases. Exercise is an effective approach recommended by current guidelines for both primary and secondary prevention of MetS and CVDs [18]. Previous evidence suggested that exercise improves lipid profiles, reduces body fatness accumulation, lower BP and blood glucose levels through its underlying pleiotropic mechanisms [25, 26]. A meta-analysis has shown that aerobic exercise led to a reduction of approximately 2 % in total cholesterol, 3 % in LDL-C, and 5 % in TG, and an increase of 3 % in HDL-C [27]. In our study, most of the cardiometabolic biomarkers were improved and HDL-C was increased pronouncedly by the 12-week moderate intensity aerobic exercise training. Similarly, Dalleck et al. found that moderate aerobic exercise (60 % of heart rate) increased HDL-C levels in healthy obese adults [28]. Additionally, we also found that athletes with higher BMI tend to have more significant changes of cardiometabolic risk factors in response to aerobic exercise intervention. We classified all athletes into two subgroups by using a BMI cutoff point of 40 kg/m^2^. This BMI cutoff point is only used for classifying their body sizes among obese athletes but not for assessing adiposity. The main purpose of such a subgroup analysis was to assess metabolic parameters among these heavy-weight class athletes with different body sizes. After we adjusted the baseline BP, the effects of exercise on SBP were still more pronounced in the high BMI group, but the difference of changes in DBP was marginally significant between these two BMI groups. It remains controversial regarding which type and intensity of exercise is optimal for cardiometabolic health. The appropriate fat-reducing exercise intensity proposed by American College of Sports Medicine (ACSM) is 40/50 to 70 % VO_2max_ [15], with a 140–170 beats/min heart rate. Aerobic exercise is a type of lower to moderate intensity physical activity (60–85 % of the maximum heart rate), which promotes increased use of oxygen in order to improve the overall body condition. Aerobic exercise promotes the activation of various anti-inflammatory functions, which, in turn, may reduce the risk of developing inflammation related chronic diseases, such as hypertension, type 2 diabetes, and cardiovascular disease [29]. Collectively, several meta-analyses of 15–54 randomized controlled trials among patients or healthy populations have suggested that ≥30 min/session aerobic exercise for 4–104 weeks provided a moderate decreasing effects on lipid levels (decrement: 2 % for TC, 3 % for LDL-C, and 5 % in women and 9 % in men for TG), weight loss (decreasing weight: 1.7 kg, circumference: 2.12 cm), and BP (−3.84 mmHg for SBP, −2.58 mmHg for DBP) [26, 27, 30–33]. Taking together, these data recommend aerobic exercise of 2–5 session/week for 20–60 continuous minutes at a moderate intensity of 40–90 % of maximum heart rate for cardiovascular health in the general population [27].

Athletes in the highest weight class usually undertake intensive strength training, the necessary amount and intensity of aerobic exercise to improve their cardiometabolic health has not been described. Some evidence suggested that aerobic exercise was more beneficial than resistance exercise on improving glycemic control in patients with type 2 diabetes; glycosylated hemoglobin levels of patients with aerobic exercise were reduced by 1.97 mmol/mol as compared to resistance exercise [8]. Nevertheless, lack of sufficient evidence to confirm the clinical importance of these statistical improvements [8, 34, 35]. The findings from our pilot trial provided suggestive evidence that aerobic exercise might provide additional cardiovascular beneficial effects among professional athletes who already received of intensive strength training. Since all participants were professional strength-trained athletes, our results may not be generalizable to other athletes or the general population. Due to our pilot trial design without randomly assigned comparison groups, our study cannot assess the cardiometabolic effects by aerobic exercise versus strength exercise. Future long-term comprehensive studies focusing on the appropriate types and intensity of exercise are warranted.

Several limitations in the present study should be considered. First, this trial was not a randomized controlled trial and lacked randomization and blind design, which can allow for minimizing bias and confounding over the trial duration. Second, the 12-week duration is relatively short. The results cannot be drawn on the long-term effect of aerobic exercise, although we observed a trend towards an improvement in MetS risk factors. Third, the sample size of 49 athletes was small to provide sufficient power for detecting a clinically significant transition of MetS and exploring potential effect modifiers. Fourth, there may exist residual confounders such as lifestyle and dietary status, although lifestyle and dietary patterns were similar because the professional Chinese athletes enrolled in this study lived in closed and standard training facilities with a strict ban on cigarette smoking and alcohol drinking. Fifth, there was lack of more accurate measures to evaluate body composition (body fat and lean body mass) and some metabolic biomarkers, such as those of adipokines and systemic inflammation. Sixth, we used Cooper 12-min running test to indirectly evaluate the capacity of aerobic exercise although this test is not an optimal measurements for capacity of exercise. Seventh, we only tested absolute HR value 5 min after exercise and did not test 1 min heart rate recovery. Finally, we assumed that baseline levels of exercise training of athletes were equivalent, although there may have some differences of regular training in intensity and amounts among these athletes in the highest weight class.

## Conclusion

In summary, our pilot trial showed that a 12-week supervised moderate intensity aerobic exercise training provided additional beneficial effects on cardiometabolic risk factors and exercise capacity among 49 metabolically abnormal athletes in the heaviest weight class who received intensive strength training. Our findings indicate that aerobic exercise training could be considered as an effective model for improving cardiometabolic health in professional athletes, especially those at high risk, although future long-term and large-scale randomized controlled trials with well-designed exercise modalities are warranted to evaluate long-term cardiovascular effects of exercise.

## Abbreviations

MetS: metabolic syndrome
BMI: body mass index
WHR: waist-to-hip ratio
CVDs: cardiovascular diseases
HDL-C: high-density lipoprotein cholesterol
LDL-C: low-density lipoprotein cholesterol
BP: blood pressure
SBP: systolic blood pressure
DBP: diastolic blood pressure
VO_2peak_: peak oxygen uptake
NCEP ATP III: National Cholesterol Education Program Adult Treatment Panel III
CVs: coefficients of variations
TG: triglyceride
ACSM: American College of Sports Medicine
VO_2max_: maximal oxygen uptake
SD: standard deviations
